# An overview of osteopathy graduates’ perceived preparedness at transition from educational environment to clinic environment one year after graduation: a cross sectional study

**DOI:** 10.1186/s12909-018-1429-2

**Published:** 2018-12-22

**Authors:** E. Luciani, G. Consorti, P. L. S. van Dun, O. Merdy, C. Lunghi, M. Petracca, J. E. Esteves, F. Cerritelli

**Affiliations:** 1 Clinical-based Human Research Department, Research Division, COME Collaboration, Pescara, Italy; 2 Research Division, COME Collaboration, Mechelen, Belgium; 3 British School of Osteopathy (BSO), London, UK; 4 Institut des Hautes Etudes Ostéopathiques (IdHEO), Saint-Herblain, France; 5 Centro Ricerche Olistiche per la Medicina Osteopatica e Naturale (CROMON), Rome, Italy; 6 Centre pour l’Etude, la Recherche et la Diffusion Osteopathiques (CERDO), Rome, Italy; 7 Instituto Piaget, Lisbon, Portugal; 8 Società Italiana di Pedagogia Medica (SIPeM), Via Capitinzano 33, 00178 Rome, Italy; 9 I.F.O.P. Chinesis, Rome, Italy

**Keywords:** Preparedness, Graduates, Survey, Education, Regulation, Europe, Osteopathic institutions, Medical education

## Abstract

**Background:**

This study investigated perceived preparedness to practice, one year after graduation across osteopathic education institutions (OEIs) and explored possible differences between countries where osteopathy is regulated (Reg) and countries where it is not (Unreg).

**Methods:**

Two hundred forty-five graduates from 7 OEIs in 4 European countries, already assessed in a previous study, were contacted one year after their graduation to complete the survey. Survey tools included a questionnaire to assess perceived preparedness to practice: Association of American Medical Colleges (AAMC) questionnaire, and a questionnaire to collect socio-demographic information and practice characteristics.

**Results:**

One hundred sixty-eight graduates (68.6%) completed the survey. The AAMC mean score one year after the graduation (23.19; confidence interval 22.81–23.58) was significantly higher than in the previous study (17.58; 16.90–18.26) (*p* < 0.001). A difference was also found between Reg (23.49; 23.03–23.95) and Unreg (22.34; 21.74–22.94) (*p* = 0.004). Osteopaths with a previous healthcare degree scored significantly higher on AAMC score (25.53; 24.88–26.19) than osteopaths without a previous healthcare degree (22.33; 21.97–22.69) (*p* < 0.001). Regulation and a previous degree were the only significant independent variables in the most predictive multivariate linear model. The model had an r^2^ = 0.33.

**Conclusions:**

Graduates from OEIs where osteopathy is regulated felt significantly better prepared to practice than Unreg. Systematic information searches about graduates’ perception of preparedness to practice, may enable OEIs to strengthen their existing curricula to ensure their graduates are effectively prepared to practice.

**Electronic supplementary material:**

The online version of this article (10.1186/s12909-018-1429-2) contains supplementary material, which is available to authorized users.

## Background

### Preparedness

Healthcare graduates are expected to be prepared for the first day of professional practice, and although they are required to display the knowledge, skills and attitudes that enable them to effectively start their working life, several studies in the field of medical education have found that graduates feel underprepared for clinical practice [1–3]. Until recently, students’ and new graduates’ perceived preparedness to practice was under-researched in osteopathy. Preparedness may be challenging to define [4]. Indeed, in the medical field, it is considered as an immediate skills-based competency or knowledge-based issue, [5–7] while others focused on the personal/interpersonal aspects of preparedness in terms of medical student resilience [8–10]. Nevertheless there is no explicit definition of the construct of ‘preparedness’ in the osteopathic field across the European countries. A study commissioned by the General Osteopathic Council (GOsC) found that UK osteopathic graduates commonly reported gaps in business and entrepreneurial skills, and patient management skills [11]. In this study, colleagues and employers considered osteopathy graduates only partially prepared for developing effective, patient centred treatment plans and promoting self-help [11]. In Australia, Subramaniam et al. [12] found that osteopathy graduates felt underprepared in medico-legal procedures, risk management, paediatrics and prognosis and management of difficult patients. Differences in perceived preparedness were also reported among European osteopathic educational institutions (OEIs) [13, 14]. These findings are similar to those from research in the field of medical education [2, 3]; however, those studies could not give an insight in which area students felt underprepared and did not assess the risks for patients safety.

The way to assess perceived preparedness to practice is debated. Postal and online surveys, interviews studies and focus groups have been used in the field of medical education [3, 15–17]; however, there is no consensus regarding the optimal method to use. In addition, studies on medical students found that curricula [18] and ethnicity [19] may have an impact on the studies’ findings. Furthermore, gender has been found to be related to the preparedness both in medical [19] and osteopathic students [13].

In the UK, the General Medical Council (GMC) [20] acknowledges the difficulties in assessing the impact of graduates’ preparedness to practice on patient care. Moreover, self-assessment has been shown to have limited reliability [17, 21]. In osteopathy, Freeth and colleagues [11] argued that preparedness to practise can never be fully complete at the end of an osteopathy degree, because certain aspects have to occur through engagement in workplace practices. Furthermore, perceived preparedness may vary when graduates start their professional work. Subramaniam et al. [12] reported that students in the final months of their osteopathy education programme felt more competent than after 6 months in practice. To ease the transition from the educational environment to the clinical practice, the GMC suggested assistantship, shadowing and induction, while in the field of osteopathy, the GOsC is currently developing a project for mentoring new graduates [11]. Despite existing evidence from medical education research highlighting students’ perceived unpreparedness to practice, research in osteopathy is still scarce [11]. This may be in part attributed to the disparity in statutory regulation in osteopathy worldwide, and particularly in Europe. Whereas in Finland, France, Denmark, Iceland, Lichtenstein, Malta, Portugal, Switzerland and the UK the profession is statutorily regulated, and the professional bodies duty is to promote high standards of patients care, professionalism, education and training; however in many other European countries the legal framework is largely still in development. Consequently, the standards of professionalism, education and patient care are diverse, and little is known about students’ and new graduates’ levels of preparedness to practice, and osteopathic care in general.

In 2015, a study investigating the perceived preparedness of osteopathic undergraduate students [13] found higher level of preparedness and satisfaction amongst students from osteopathic institutions located in countries without regulation compared to those located in countries where osteopathy is regulated. Following this research, the aims of the present study are twofold. First, assessing perceived preparedness to practice, 1 year after graduation, in the same cohort of students assessed in the previous study, and second, to explore possible differences between graduates in countries where osteopathy is regulated and in countries where it is not.

## Methods

### Participants

Nine full-time OEIs, which participated in a previous study investigating the perceived preparedness to practice, [13] were contacted in September 2015 explaining the aim of the study.

Seven accepted to participate: four were from Italy and one from the Netherlands, where osteopathy is not yet regulated and two were respectively from France and UK, where osteopathy is regulated. Two hundred forty-five students were invited by the participating schools to fill out the questionnaire to assess their perception of preparedness 1 year after graduation. Students eligible for recruitment were those who participated in the 2015 Luciani et al’s study [13].

### Procedure

In each enrolled OEI, representative researchers contacted osteopaths who graduated in the 2014/2015 academic year. Osteopaths received an explanatory e-mail detailing the study and were informed that all data collected would be de-identified, assuring anonymity. Questionnaires were uploaded into a tailored online platform and graduates received a unique/single e-mail where a token link was embedded and allowed to access to the questionnaires. The platform allowed each e-mail address only one access, to avoid double responses related biases. A reminder was sent 3 months later, and the online access was closed 4 months after the initial invitation.

### Instrument

Perceived preparedness to practice was the main outcome of this study and it was assessed using the Association of American Medical Colleges (AAMC) questionnaire. The AAMC consists of 7 statements and was used to assess the perceived preparedness in a previous study [13]. The domains represented a wide range of competencies in seven clinical areas, summarised as follows: 1) general clinical skills, 2) basic knowledge of diagnosis and management of common conditions, 3) communication skills, 4) skills for clinical decision making and evidence-based practice to clinical care, 5) basic abilities on managing issues in medicine, 6) professionalism, and 7) basic abilities for patient care. Participants rated each of these areas using a five-point Likert scale (strongly agree = 4, agree = 3, uncertain = 2, disagree = 1, strongly disagree = 0). A higher score indicated a higher level of perceived preparedness. In addition to the AAMC questionnaire, to explore how newly graduated osteopaths considered their education, the following question was asked “Generally do you think your school prepared you well for practice (for your profession)?” (Additional file 1: Table S1).

Another questionnaire was utilized to gather general and practice characteristics and demographics. The questionnaire consisted of 50 items and was previously piloted and used in the Benelux osteosurvey study [22].

Questionnaires were translated from English to Italian for the four institutions based in Italy and from English to French for the institution based in France. A rigorous backward-forward translation process has been applied to control for linguistic and conceptual equivalence and to assure cultural adaptation.

### Statistical analyses

The score of the AAMC test was treated as a continuous variable since the distribution proved to be normal, with a kurtosis = 0.06 indicating an almost perfect bell shape. Hence the difference between the mean score of different groups was assessed with Student t-test, with a threshold of 0.05 for alpha error. Confidence intervals (CI) at 95% were computed as well. ANOVA was used to compare the mean AAMC scores of all the OEIs.

Difference in the distribution of categorical variables was assessed using a chi square test with a significance threshold less than 0.05.

The correlation between two variables was assessed with linear regression and expressed with a correlation coefficient (r).

Adjustment for covariates and possible confounding factors was done with a multivariate linear regression model, developed with a backward selection.

The only variable for which missing data were present was the work modalities: alone or group of practice. Statistics refers only to the respondents.

SPSS® and Microsoft Excel software were used for the statistical analyses.

## Results

### Sample

Two hundred forty-five newly graduated osteopaths from 7 OEIs were contacted and 168 participated at the study (response rate 68.6%).

The majority of participants were male (57.1%) and younger than 30 years (82.1%).

All respondents were self-employed, 31.5% of them worked in a group practice, 19.0% worked alone and the remaining 49.5% did not declare their work modalities. Overall, 73.2% of the respondents had not any previous healthcare degree. The 50.6% of respondents declared to work less than 17 h a week. The majority of the respondents attended less than 2 continuous professional development (CPD) events during the last year (73.8%).

An overview of all characteristics is shown in Table 1.

**Table 1:** Demographic characteristics of the participants (chi square test)

Sample	Regulated (%)	Unregulated (%)	Total (%)	*p* value
Male	67 (54.03)	29 (65.91)	96 (57.14)	0.23
Female	57 (45.97)	15 (34.09)	72 (42.86)	
Total	124	44	168 (100)	
20–29 y/o	96 (77.42)	42 (95.45)	138 (82.14)	0.01
30–39 y/o	28 (22.58)	2 (4.55)	30(17.86)	
Group of practice	46 (70.77)	7 (35.00)	53 (31.5)	0.009
Alone	19 (29.23)	13 (65.00)	32 (19.0)	
Healthcare degree	37 (29.84)	8 (18.18)	45(26.79)	0.19
Healthcare degree	87 (70.16)	36 (81.82)	123 (73.21)	
< 17 h/w	72 (58.06)	13 (29.55)	85 (50.60)	0.59
> 16 h/w	52 (41.94)	31 (70.45)	83 (49.40)	
< 3 cpd	100 (80.65)	24 (54.55)	124 (73.80)	0.001
> 2 cpd	24 (19.35)	20 (45.45)	44(26.20)	

### AAMC scores

The mean AAMC score 1 year after graduation was 23.19 (standard deviation: 2.52, minimum: 17, maximum: 28, median: 23, kurtosis: 0.06). A small but significant difference was found 1 year after graduation between Reg (23.49; CI 23.03–23.95) and Unreg (22.34; CI 21.74–22.94) (*p* = 0.004). Respondents with a previous healthcare degree had a significantly higher AAMC score (25.53; CI 24.88–26.19) than respondents without a previous healthcare degree (22.33; CI 21.97–22.69) (*p* < 0.001). Respondents who practiced alone achieved a significantly higher mean AAMC score (24.19; CI 23.14–25.24) than those who practiced in a group practice (22.81; CI 22.25–23.38) (*p* = 0.03).

The AAMC mean scores are shown in Table 2.

**Table 2:** AAMC scores (Student t test)

Sample	Mean, SD	CI	p
Undergraduate*	17.58, 5.38	16.90–18.26	< 0.001
1 year of practice	23.19, 2.52	22.81–23.58	
Regulated	23.49, 2.62	23.03–23.95	0.004
Unregulated	22.34, 2.02	21.74–22.94	
Regulated*	16.70, 11.00	na	< 0.001
Regulated (present study)	23.49, 2.62	23.03–23.95	
Unregulated *	20.56, 5.32	na	0.02
Unregulated (present study)	22.34, 2.02	21.74–22.94	
20–29 y/o	23.31, 2.63	22.88–23.76	0.1
30–39 y/o	22.63, 1.89	21.96–23.30	
Female	23.00, 2.16	22.50–23.50	0.38
Male	23.33, 2.85	22.78–23.89	
Healthcare Degree	25.53, 2.25	24.88–26.19	< 0.001
Non Healthcare Degree	22.33, 2.03	21.97–22.69	
Group of practice	22.81, 2.09	22.25–23.38	0.03
Alone	24.19, 3.03	23.14–25.24	
< 3 cpd	23.16, 2.41	22.74–23.59	0.34
> 2 cpd	23.63, 2.72	22.77–24.49	
< 17 h/w	23.09, 2.12	22.64–2354	0.62
> 16 h/w	23.29, 2.89	22.67–23.91	

*Data are derived from [13]

A very weak but significant correlation was found (*r* = 0.193; p = 0.03) between the AAMC score and the score of the added item related to the perception of how well the OEI prepared students to practice. The mean score of this item was also significantly different between Reg and Unreg (3.53 vs 2.73; *p* < 0.001).

Regulation and a previous degree were the only significant independent variables in the most predictive multivariate linear model (Table 3). The model had an r^2^ = 0.33.

**Table 3:** Multivariate linear model

	Beta	Std. Err.	t	Sign.
(Constant)	17.878	0.757	23.610	0.0001
Reg vs Unreg	0.789	0.366	2.157	0.032
Healthcare degree Vs Not Healthcare degree	3.109	0.363	8.567	0.0001

Dependent Variable: AAMC score; r^2^ = 0.33

A significant difference was found in mean scores of four out of seven AAMC items (Table 4) between Reg and Unreg. Reg scored higher than Unreg on items three (3.19 vs 2.77; *p* < 0.001), four (3.57 vs 3.30; *p* = 0.005) and five (3.43 vs 3.16; *p* = 0.04). However, the average score of item seven showed a significant difference between Reg and Unreg in favor of Unreg (3.23 vs 3.48; *p* = 0.01).

**Table 4:** AAMC items score comparison: regulated vs uregulated

Item	Group	Mean, SD	p
I am confident that I have acquired the clinical skills required to work as an osteopath	Item 1(regulated) Item 1 (unregulated)	3.26, 0.64 3.00, 1.89	0.08
I believe I have the fundamental understanding of common conditions and their management encountered in the major clinical disciplines	Item 2 (regulated) Item 2 (unregulated)	3.14, 0.70 3.11, 0.49	0,80
I have the communication skills necessary to interact with patients and health professionals	Item 3 (regulated) Item 3 (unregulated)	3.19, 0.67 2.77, 0.64	< 0.001
I have basic skills in clinical decision making and the application of evidence based information to osteopathic practice	Item 4 (regulated) Item 4 (unregulated)	3.57, 0.57 3.30, 0.55	0.006
I have a fundamental understanding of the issues in social sciences of osteopathic medicine (e.g., ethics, humanism, professionalism, organization and structure of the health care system)	Item 5 (regulated) Item 5 (unregulated)	3.43, 0.60 3.16, 0.78	0.04
I understand the ethical and professional values that are expected of the profession	Item 6 (regulated) Item 6 (unregulated)	3.69, 0.47 3.52, 0.50	0.06
I believe I am adequately prepared to care for patients from different backgrounds	Item 7 (regulated) Item 7 (unregulated)	3.23, 0.80 3.48, 0.50	0.02

Finally, a significant difference in mean AAMC score was detected among the participating schools (*p* < 0.001) (Table 5).

**Table 5:** AAMC mean score of the participating OEIs

OEIs	n. of students	Sum	Mean	Variance	
School 1	7	146	20.86	0.81	
School 2	42	922	21.95	1.85	
School 3	13	306	23.54	2.27	
School 4	13	282	21.69	4.06	
School 5	11	231	21.00	3.60	
School 6	75	1845	24.60	7.08	
School 7	7	164	23.43	1.95	
	*SQ*	*DoF*	*MS*	*F*	*p*
Among groups	335.43	6	55.90	12.36	< 0.001
In groups	728.48	161	4.52		
Total	1063.91	167			

## Discussion

Investigating students’ perception of their preparedness to practice can provide opportunities to improve the quality of education.

This study investigated perceived preparedness to practice, 1 year after graduation across seven European OEIs. Our results showed that perception of preparedness increased after 1 year of practice in the same cohort assessed in a previous study (Table 2) [13]. This result is in agreement with Subramaniam et al. [12] who found that Australian osteopathic graduates felt more competent after 6 months in practice, even if this study was limited by its low response rate (27%). Interestingly, in the present study no significant difference was found between male and female osteopaths as to overall perceived preparedness. This is in contrast with our previous study [13] where being female was associated with a change in the perception of being prepared to practice.

Osteopaths from both countries with statutory regulation and osteopaths from countries with no statutory regulation significantly improved their perceived preparedness compared with our previous study (Table 2) [13]. Nevertheless, in the previous study Unreg scored higher than Reg, while in the present study Reg scored significantly higher than Unreg (Table 2). A possible explanation for this inversion is that after 1 year of professional practice, the Reg are likely to be integrated within their respective societies private or public healthcare services. The lack of a formal support from the healthcare community may influence the self-confidence of the osteopaths and consequently their perceived preparedness.

Graduates’ preparedness may be affected by differences in curriculum design and educational strategies, [15, 17] learning environment, [1, 23] class size [13, 24] and greater exposure to clinical practice [25]. In this study no statistical significant association was found between the number of hours of practice and respondents’ perceived preparedness.

Preparedness to practice may also be affected by holding a previous degree, i.e., a past higher education experience may enhance preparedness [26]. This is in agreement with the results of the present study in which the respondents who had a previous healthcare education, scored significantly higher on the AAMC. It can be argued that having another degree may enhance cognitive and critical thinking skills required to effectively operate as a primary contact healthcare professional dealing with situations of clinical uncertainty [27, 28]. Considering age, Surmon et al. [29] stated that prior life experiences rather than age itself that was the underlying factor that could impact on preparedness. This last finding is in agreement with our results because in our data perception of preparedness was not related to age.

Considering the significantly higher perceived preparedness of respondents who work in a group practice, it could be argued that, working in a group of practice exposes osteopaths to a wider variety of clinical cases and discussions, increasing their opportunities to self-reflect on knowledge and skills gaps relating to aspects of their clinical work. Nevertheless the inter/intra-professional interaction they experience could strengthen their preparedness, independently from their perceived preparedness [30].

The most predictive multivariate linear regression model showed that regulation and a previous degree were the only significant independent variables accounting for the 33% of the variance. This finding is consistent with the complexity of the perceived preparedness construct which showed to be multi-factorial [4]. Moreover, preparedness and perceived preparedness are seen as two different constructs [4]. To our current knowledge there are no studies which have investigated the relationship between these two constructs.

We found a very weak but significant correlation between the AAMC score and the score of the added item exploring the perception of how well the OEI prepared students to practice. The mean score of this item was also significantly higher for Reg than for Unreg osteopaths. This perceived lower preparedness for Unreg is consistent with our data showing that Unreg attended more CPD events than Reg (Table 1). However it is unclear how the “OEI perceived preparation to practice” construct was intended and assessed by the respondents. The construct validity of this item should be further investigated in order to understand how to interpret it.

### Considerations on AAMC specific items

The single item analysis of the AAMC could be of interest to adjust the educational focus on a specific field. Our results showed a significant difference between Reg and Unreg on items: communication skill, clinical decision-making skills, social science fundamentals, preparedness to take care of patients from different backgrounds. These topics could be grounds for international strategies aimed to improve the international osteopathic education level. Strategies to increase the perceived preparedness of students have been explored in relation to healthcare education. For example, shadowing and mentoring are used to increase students perceived preparedness. During assistantship students assist junior practitioner and undertake most of the duties of a practitioner under supervision. Studies showed that assistantship increased perception of preparedness to start to work in students [31, 32]. Therefore, after graduation new graduates undertake a period of shadowing where the new practitioner assists the more experienced practitioner as peers before taking their place. We argue that similar strategies could enhance preparedness to practice in osteopathy, particularly in the initial post-qualifying year in osteopathic practice.

## Limitations

This study has several limitations. Firstly, self-assessment method has its own limitation regarding reliability [17, 21]. Moreover, it can be argued that measuring the perception of preparedness using a 7-question tool, although validated and largely used in the medical education field, might reflect *confidence* to practice rather than *competence*. In fact, the latter needs to be assessed using more complex, specific and holistic qualitative and quantitative instruments. Secondly, a bias could have been introduced since 31.4% of the original cohort did not participate to the survey. This could have influenced the results. Another limitation is that it is not possible to determine if our sample is representative of the entire population of European osteopathic students as lack of formal data on the topic.

## Conclusions

Respondents graduated from OEIs in countries with statutory regulation felt significantly better prepared to practice than their colleagues from countries without regulation. A possible interpretation of this finding is that not being integrated within the national healthcare system or regulation bodies and the lack of a formal support from the healthcare community could influence the self-confidence of the osteopaths and consequently their perceived preparedness. This hypothesis should be confirmed by further research. Systematic information about graduates’ perception of preparedness to practice may enable OEIs to strengthen their existing curricula to ensure their graduates are effectively prepared to practice.

## Additional file

Additional file 1: **Table S1.** Details on number of questions and description regarding Association of American Medical Colleges (AAMC) questionnaire. (DOCX 17 kb)

## Abbreviations

AAMC: Association of American Medical Colleges
CPD: Continuous professional development
GMC: General Medical Council
GOsC: General Osteopathic Council
OEIs: Osteopathic education institutes
Reg: Countries where osteopathy is regulated
Unreg: Countries where osteopathy is not regulated
