# Supplementary material for: An overview of osteopathy graduates’ perceived preparedness at transition from educational environment to clinic environment one year after graduation: a cross sectional study
Source: BMC Med Educ. 2018 Dec 22;18:319. doi: 10.1186/s12909-018-1429-2 (PMC6303955; doi:10.1186/s12909-018-1429-2)
Supplement: Supplementary file 1 — Table S1. Details on number of questions and description regarding Association of American Medical Colleges (AAMC) questionnaire. (DOCX 17 kb) [file 12909_2018_1429_MOESM1_ESM.docx]

Additional file 1

Table S1. Details on number of questions and description regarding Association of American Medical Colleges (AAMC) questionnaire.

| Number | Description |
| --- | --- |
| 1 | I am confident that I have acquired the clinical skills required to work as an osteopath |
| 2 | I believe I have the fundamental understanding of common conditions and their management encountered in the major clinical disciplines |
| 3 | I have the communication skills necessary to interact with patients and health professionals |
| 4 | I have basic skills in clinical decision making and the application of evidence based information to osteopathic practice |
| 5 | I have a fundamental understanding of the issues in social sciences of osteopathic medicine (e.g., ethics, humanism, professionalism, organization and structure of the health care system) |
| 6 | I understand the ethical and professional values that are expected of the profession |
| 7 | I believe I am adequately prepared to care for patients from different backgrounds |

Question added to AAMC: Generally do you think the school prepared you well for practice (for your profession)?
